# Non‐Reciprocity, Metastability, and Dynamic Reconfiguration in Co‐Assembly of Active and Passive Particles

**DOI:** 10.1002/advs.202409489

**Published:** 2024-12-04

**Authors:** Ahmed Al Harraq, Ruchi Patel, Jin Gyun Lee, Ope Owoyele, Jaehun Chun, Bhuvnesh Bharti

**Affiliations:** ^1^ Cain Department of Chemical Engineering Louisiana State University Baton Rouge LA 70803 USA; ^2^ Center for the Physics of Biological Function Princeton University Princeton NJ 08544 USA; ^3^ Department of Chemical and Biological Engineering University of Colorado Boulder CO 80303 USA; ^4^ Department of Mechanical and Industrial Engineering Louisiana State University Baton Rouge LA 70803 USA; ^5^ Physical and Computational Sciences Directorate Pacific Northwest National Laboratory Richland WA 99354 USA

**Keywords:** active colloids, colloidal assembly, electric field, metastable assembly, patchy particles

## Abstract

Living organisms often exhibit non‐reciprocal interactions where the forces acting on the objects are not equal in magnitude or opposite in direction. The combination of reciprocal and non‐reciprocal interactions between synthetic building blocks remains largely unexplored. Here, out‐of‐equilibrium assemblies of non‐motile isotropic passive and metal‐patched motile active particles are formed by overlapping bulk interactions with directed self‐propulsion. An external alternating current (AC) electric field generates concurrent dipolar and induced‐charge electrophoretic forces between the particles which are evaluated using microscopy. The interaction force measurements allow to determine the degree of reciprocity in interactions, which is tunable by designing the active particle and its trajectory. While linearly‐propelled active particles evade assembly with passive particles, helically propelled active particles form active‐passive clusters with dynamic reconfiguration and long‐lived metastability. Large clusters display programmable fluctuations and reconfigurability by controlling the fraction of active particles. The study establishes principles of integrating reciprocal and non‐reciprocal interactions in guided colloidal assembly of reconfigurable metastable structures.

## Graphical abstract

This study investigates the interactions between surface isotropic and patchy particles in AC electric fields. The induced‐charge electrophoresis of Janus‐type particles introduces non‐reciprocity in the field‐driven interaction with other non‐motile particles. Here, the degree of non‐reciprocity is measured, and active‐passive interactions are modulated by varying patch morphology to form otherwise inaccessible motile colloidal structures.

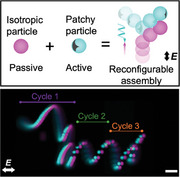

## 1 Introduction

Active colloidal particles differ from common passive particles in their ability to convert external energy into directed motion.^[^ ^1^, ^2^, ^3^ ^]^ This property, which is normally attributed to biological swimmers such as bacteria, underlies the development of nano‐ and microscale motors.^[^ ^4^, ^5^, ^6^ ^]^ The last two decades have witnessed the design of active particles driven by a variety of energy sources such as chemical,^[^ ^7^, ^8^, ^9^, ^10^ ^]^ electrical,^[^ ^11^, ^12^, ^13^ ^]^ magnetic,^[^ ^14^, ^15^, ^16^ ^]^ optical,^[^ ^17^, ^18^, ^19^ ^]^ and acoustic.^[^ ^20^, ^21^, ^22^ ^]^ The augmented dynamics of these self‐propelled particles compared to their passive counterparts lend themselves to robotic miniaturization and the bottom‐up assembly of colloidal devices.^[^ ^23^, ^24^, ^25^, ^26^ ^]^

One major challenge in the field of active colloids is to achieve control over the microstructure of an assembly while retaining the activity of the individual components. Interactions involving active particles are non‐reciprocal, i.e., they appear to break Newton's third law of action‐reaction.^[^ ^27^, ^28^, ^29^, ^30^, ^31^, ^32^, ^33^ ^]^ This occurs as, unlike bulk interactions, fluid‐mediated interactions between particles are dissipated in the surrounding environment. For example, hydrodynamic interactions between two microswimmers are not pairwise mutual but effectively multi‐body such that the net forces on the microswimmers are not equal or opposite. Non‐reciprocal interactions can give rise to life‐like behaviors such as predator‐prey dynamics^[^ ^27^ ^]^ as well as parasitic^[^ ^34^ ^]^ and commensal pairings.^[^ ^35^ ^]^ In the case of artificial active particles, non‐reciprocity can be a hindrance to the stable formation of colloidal assemblies. Instead, the hydrodynamics of active particles often lead to transient forms of collective organization such as motility‐induced phase separation and clustering which occur at high particle concentration.^[^ ^36^, ^37^ ^]^ By contrast, stable colloidal assemblies generally require bulk reciprocal interactions which can impart well‐defined structural motifs.^[^ ^38^, ^39^, ^40^, ^41^ ^]^

Efforts to structure active colloidal materials must contend with the dichotomy between achieving long‐lived metastability^[^ ^42^, ^43^ ^]^ while enabling motility and spontaneous reconfiguration.^[^ ^44^, ^45^ ^]^ Solving this dichotomy underscores the design of active colloidal structures that possess a “bulk” structural motif while the building blocks are free to individually respond to changes in the energy landscape. One approach to tackle this is to combine directional and programmable bulk interactions with a motility‐driven component.^[^ ^46^, ^47^, ^48^ ^]^ This superposition of reciprocal and non‐reciprocal interactions was recently theorized to unlock a design space of “shape‐morphing” assembly that lies between static equilibrium self‐assembly and transient activity‐driven self‐organization.^[^ ^45^ ^]^ However, experimental realization of such reconfigurable assemblies of colloids interacting via overlapping reciprocal and non‐reciprocal interactions has remained elusive, and is the primary focus of this study.

**Figure 1:**
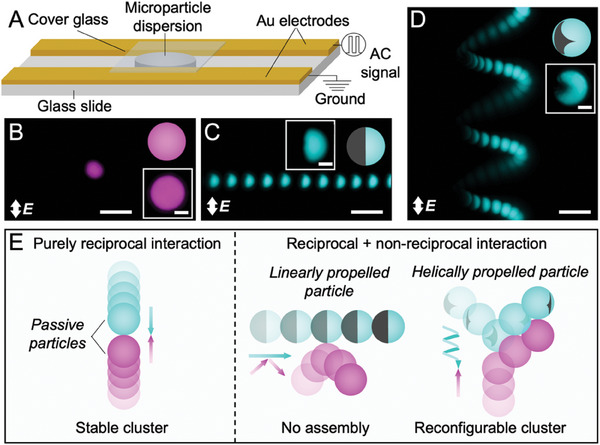
A model system for reciprocal and non‐reciprocal interactions in AC electric fields. A) Schematic of the coplanar Au electrode setup used to apply the AC electric field. The microparticle dispersion is placed on the glass slide between the electrodes and sandwiched with a cover glass. An AC external electric field of frequency 10 kHz (square‐wave) and target strength in the range 10–700 V cm^−1^ is applied to the dispersion. The structure and dynamics of the microparticles are visualized in the plane containing the electrodes using optical microscope in brightfield and fluorescence modes. B–D) Overlays of the fluorescence micrographs showing the trajectories of (B) isotropic, (C) Janus, and (D) chiral triangular patchy particles in AC electric field. The images are generated by overlaying the individual frames of Video S1 (Supporting Information) with 3 s elapsed time for the isotropic and Janus particle and 5 s for the triangular patched particle. The insets show high‐magnification fluorescence micrographs of the respective particles where the dark regions on the particles represent the Au patches. The applied field strength in (B–D) is 300 V cm^−1^ at 10 kHz. Scale bars in (B–D) are 10 µm and in insets are 2 µm. E) Conceptual summary of the transition from reciprocal dipolar assembly between two passive particles, to the combination of reciprocal and non‐reciprocal interaction leading to deflection or assembly between passive and active particles in AC electric field.

In this article, we use AC electric fields to investigate the interactions of a mixture of active and passive particles. To achieve this, we combine a set of magenta polystyrene microspheres (radius, *R* = 2.6 µm) with a set of equally‐sized cyan polystyrene microspheres. Both magenta and cyan particles have negative surface charge and nearly identical physicochemical characteristics. In all experiments, the magenta particles are isotropic whereas the cyan particles are either also kept isotropic or equipped with a thin gold (Au) patch (thickness 30 nm). An aqueous suspension of these particles is placed on a coplanar electrode setup which we use to apply the AC electric field, ***E*** (**Figure** **1**A). All experimental details can be found in the Experimental section.

Particles exposed to the AC electric field become polarized due to shearing of their electrical double layer^[^ ^49^ ^]^. This shearing of the low polarizability isotropic particle surface leads to weak symmetric induced charge electroosmotic (ICEO) flows without any significant self‐propulsion, i.e., isotropic particles in AC electric fields are nearly passive (Figure 1B; Figure S1, Supporting Information). Conversely, Au patches are more strongly polarized causing imbalance in ICEO flow around the particle leading to their propulsion known as induced‐charge electrophoresis (ICEP),^[^ ^50^, ^51^ ^]^ i.e., patchy particles in AC electric fields are active (Figure S1, Supporting Information). The shape of the patch determines the trajectory of motion such that Janus particles comprising hemispherical Au patches propel linearly (Figure 1C). We previously reported that particles comprising smaller triangular patches propel in helical trajectory (Figure 1D).^[^ ^11^, ^12^ ^]^ Dynamics of passive, linearly‐propelled, and helically‐propelled particles can be viewed in Video S1 (Supporting Information). Regardless of the motility characteristics, all particles used in the study gain a field‐induced dipole moment aligned with ***E***. Additional information on dipole‐dipole interparticle interactions and ICEP in AC electric fields can be found in Notes S1 and S2 (Supporting Information).

## 2 Interaction Dynamics Between Pairs of Active and Passive Particles

The versatility of AC electric fields allows us to program the interaction among active and passive particles and probe the corresponding collective dynamics (Figure 1E). We investigate and compare the spatiotemporal evolution of the interaction of a passive particle paired with 1) another passive particle, 2) a linearly‐propelled active particle translating orthogonal to the field direction, 3) a helically‐propelled active particle translating along the field direction and rotating in the orthogonal plane. Unless otherwise specified, we carry out all experiments with an AC electric field of 300 V cm^−1^ at 10 kHz frequency and observe the particle dynamics at up to 300 frames per second to access millisecond temporal resolution. We use the Trackmate plugin^[^ ^52^ ^]^ within ImageJ^[^ ^53^ ^]^ to obtain the change in position vectors for two interacting particles over time in a global reference frame (Figure S2, Supporting Information). Before further using the data to compute the derivatives of the position vectors, we process it using low‐bandpass filtering to maximize signal‐to‐noise ratio (see Experimental section and Note S3, Supporting Information). For each particle, we then extract the net change in speed defined as Δ*v*(*s,t*) *= v_t_ – v_o_* where *v_t_* is the particle speed at time *t* at short interparticle distances, where the interaction forces are significant, and *v_o_* is the average speed of the particle at large interparticle distances, i.e. *s/R* ≥ 1.5. We then calculate the net change in interaction forces acting on the individual particles, Δ*F*, using the mobility relation Δ*F*(*s,t*) = ‐6π*ηR*Δ*v*(*s,t*) where *η* is the viscosity of water. While measuring *v_o_* at significantly larger separations would further minimize potential artifacts, constraints on the data transfer rate during high‐frame‐rate image acquisition make such measurements impractical in our experiments.

**Figure 2:**
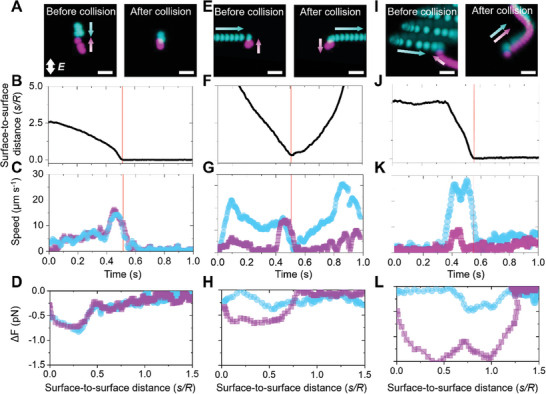
Measurement of the particle dynamics in an interacting pair. Overlayed fluorescence micrographs showcasing the motion of a passive particle (magenta) with a second particle (cyan) that is either (A) passive, (E) linearly‐propelled or (I) helically‐propelled. The applied field strength in all three cases is 300 V cm^−1^ at 10 kHz. The arrows indicate the direction of motion and scale bars in (A,E,I) are 10 µm. B,F,J) Change in surface‐to‐surface distance with time. C,G,K) Change in speed of each member of a pair of interacting particles. The red vertical lines indicate the time of collision. D,H,L) Change in net forces acting on each member of an interacting pair of particles as a function of surface‐to‐surface distance *s* normalized by particle radius *R*. Values are computed using Δ*F*(*s*,*t*) = −6𝜋*ηR*Δ*v*(*s*,*t*) with the incorporated change in net speed of the interacting particles. B) Two passive particles approach each other at similar rates until the surface‐to‐surface distance drops to zero at collision and (C) maintain equivalent speeds throughout the interaction. D) The change in net forces experienced by two passive particles are equivalent throughout the interaction. F) A linearly‐propelled particle (cyan) and a passive particle (magenta) approach each other until the surface‐to‐surface distance drops to zero at collision and increases post‐failure of assembly. G) The active particle is faster than the passive particle which displays speed fluctuations throughout the interaction. H) At *s*/*R* > 0.75, the change in net force of both the particles is zero indicating that they are force‐free. At *s*/*R* < 0.75 the net forces do not overlap indicating non‐reciprocity of the interaction. J) A helically‐propelled particle (cyan) and a passive particle (magenta) approach each other until the collision instant where the interparticle separation drops to zero and remains constant thereafter. K) The helically‐propelled particle initially moves at a higher speed which is reduced upon collision and assembly with the passive particle. L) The change in net forces acting on the helically propelled and passive particle are zero at *s*/*R* > 1.25 and diverge as *s*/*R* < 1 due to presence of significant hydrodynamic forces.

All observed pair interactions display unique characteristics yet have representative features. Two passive particles do not generate any significant fluid flow; thus, their interaction is primarily governed by dipole–dipole interactions. Typically, these particles tend to mutually align along the field axis, approaching pole‐to‐pole until they collide and assemble into a pair cluster (**Figure** **2**A; Video S2, Supporting Information) which remains stable for as long as the external field is maintained. The signature dynamics of these dipolar interactions appear within *s*/*R* ≈ 1 and in the timespan of 300 ms. Both particles in a passive‐passive pair move toward their collision point until the surface‐to‐surface distance drops to zero (Figure 2B), and experience mutual gains and losses in speed as they interact to form an assembled doublet (Figure 2C; Figure S3, Supporting Information). As a result, the Δ*v* and thus the Δ*F* measured throughout the interaction for each passive particle are nearly identical, highlighting the reciprocity in the bulk dipolar attraction (Figure 2D).

Replacing one of the passive particles in the interacting pair with an active particle changes these dynamics because of electric field‐driven fluid flows at the metal patch resulting in directional and highly localized fluid‐mediated interactions. The oscillating nature of the AC electric field causes a temporal acceleration of the surrounding fluid and the electrical double layer.^[^ ^54^, ^55^ ^]^ This gives rise to unsteady flows that manifest a “transient” drag force characterized by vorticity diffusion that may influence the interaction dynamics (Note S4, Supporting Information). The unsteady nature of the fluid flow in the vicinity of the Au patch can lead to non‐reciprocal interactions that depend on the approach trajectory, resulting from the history dependence of the transient drag force (Note S4, Supporting Information).

Linearly‐propelled particles consistently fail to form stable pairs with passive particles (Figure 2E; Video S2, Supporting Information). The active particle moves faster in the plane and, as it approaches the passive particles, it moves toward its pole. This passive‐active interacting pair appears to form a doublet that disassembles between ≈0.5 and 1.0 s as marked by the increase in surface‐to‐surface distance after the collision instant. (Figure 2E,F,G). Extracting Δ*F*(*s*,*t*) from the interaction dynamics of these passive‐active pairs shows that the superposition of dipolar with ICEP forces gives rise to a net non‐reciprocal interaction (Figure 2H). Overall, these findings show that pairs of a passive and a linearly‐propelled particle cannot achieve a stable assembled state, forming transient doublet clusters that are broken apart quasi‐instantaneously. Finite element calculations of the coupled electric and electroosmotic flow fields provide qualitative evidence that, by dictating the localization of electroosmotic flows, the shape of the patch is a key determinant of the stability of interacting pairs (discussed in next section and Note S5, Supporting Information). The Janus morphology of the linearly propelled particles is associated with strong ICEO flows at the poles, which obstruct pole‐to‐pole assembly.

## 3 Quantification of Interaction Non‐Reciprocity and Metastability

The shape of the Au patch determines the trajectory of motion which also significantly impacts the assembly of clusters by altering the non‐reciprocal component of the interaction, coupled with the unique nature of the transient drag force.^[^ ^55^ ^]^ By tuning the Au patch from hemispherical to a smaller triangular configuration, we produce active particles that display interaction dynamics that lie between the passive‐passive and linearly propelled‐passive cases. In fact, helically propelled particles form metastable doublets upon collision with passive particles which are either short‐ or long‐lived. In the former case, the clusters quickly disassemble, whereas the long‐lived clusters remain assembled and propel as motile units as long as the AC electric field is present (Figure 2I and Video S2, Supporting Information). Within these long‐lived metastable clusters, helically propelled particles appear to maintain active dynamics as they revolve on the surface of passive particles, i.e. the active particle itself rotates on the passive particle while maintaining the cluster motility (Figure 2J,K; Figure S3, Supporting Information). The extracted Δ*F*(*s*,*t*) indicates that interactions between such helically propelled active particles and corresponding passive particles are non‐reciprocal (Figure 2L) yet allow tunable pair assembly with bulk dipolar attraction. We expect that the reduction of patch size and the relocation of the intense ICEO flows away from the poles render these motile clusters more favorable compared with the linearly propelled‐passive pairs (Note S5, Supporting Information).

**Figure 3:**
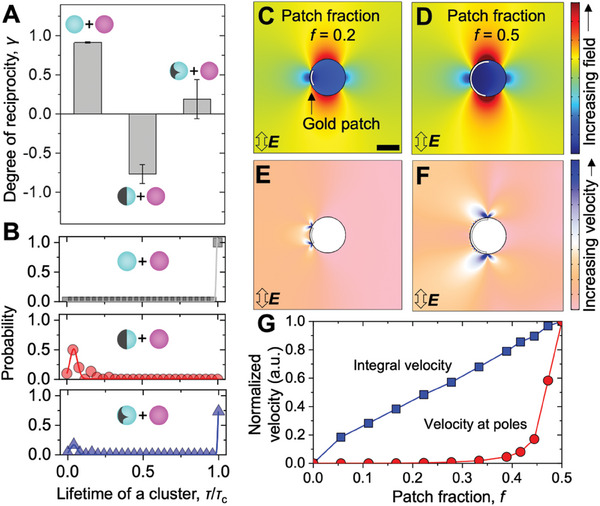
Non‐reciprocity, metastability and mechanism of formation of active doublets in AC electric field. A) Degree of non‐reciprocity in doublets formation quantified by the correlation coefficient between the magnitudes of the change in forces acting on the individual particle in the interacting pair. B) Experimentally determined probability distribution of the duration of assembly i.e., lifetime of the clusters for passive‐passive, passive‐linearly propelled, and passive‐helically propelled pairs. Here, the lifetime is normalized by the critical lifetime of the cluster which is the average time an active particle takes to move a distance of one hundred times its radius. The applied field strength for all experiments shown in (A,B) is 300 V cm^−1^ at 10 kHz. C,D) Electric field distribution and corresponding (E,F) fluid flow around a particle with a patch fraction *f* of 0.2 (left) and 0.5 (right). Scale bar in (C) is 2 µm. G) Increase in normalized velocity of the fluid computed at the polar region 0.1 µm away from the apex (red circles) and the integral velocity along the circumference 0.1 µm away from the surface of the particle (blue squares) as a function of patch fraction, *f*.

Small variations in the particle characteristics can have large impacts on the reciprocity of the pair interaction. To quantify this, we define the average degree of reciprocity, *γ*, as the correlation coefficient between the change in forces on two interacting particles in the region *s*/*R* ≤ 1. Mathematically, $\gamma =\frac{1}{n-1}\sum_{i=1}^{n}\left(\frac{\Delta F_{1,i}-\Delta {\bar{F}}_{1}}{\sigma_{1}}\right)\left(\frac{\Delta F_{2,i}-\Delta {\bar{F}}_{2}}{\sigma_{2}}\right)$, where *n* is the number of separation points for which the net forces are calculated, Δ*F*_1,*i*_ and Δ*F*_2,*i*_ are the net changes in the interaction forces acting on the particle pair at a given separation, with respective mean and standard deviation of $\Delta {\bar{F}}_{1}$ and $\Delta {\bar{F}}_{2}$; and σ_1_and σ_2_. Values of *γ* →1 correspond to purely reciprocal interactions and *γ* → −1 indicates perfect non‐reciprocity. For each pair interaction, we compute *γ* from triplicate measurements of *F_d_* at fixed electric field parameters (**Figure** **3**A). Passive–passive pairs interact with *γ* = 0.92 ± 0.01 where the uncertainty is the standard deviation, highlighting the high degree of reciprocity with minimal variability across experiments. By contrast, linearly propelled‐passive pairs interact with *γ* = −0.76 ± 0.12 indicating the highly non‐reciprocal nature of the interaction and moderate variability for each interaction. Helically propelled‐passive pairs interact with *γ* = 0.19 ± 0.25 which indicates a fine balance of the contributions of reciprocal and non‐reciprocal interactions where the operational forces on the interacting pair are statistically uncorrelated. Additionally, we find that the interactions between two passive particles of different sizes remain reciprocal as the forces induced by the external field is symmetric. However, interactions between an active and passive particle of different sizes or between two active particles with the same or different patch sizes are non‐reciprocal (Figure S4, Supporting Information). Note that in all the reported measurements, an interacting particle pair was ≈20 µm away from the substrate. Hence the observed reciprocal/non‐reciprocal relation in the interparticle interaction is not due to the presence of a third body, but a result of the energy dissipation in the mediating fluid. The addition of a non‐reciprocal component pushes the assembled states away from equilibrium, driving reconfiguration of the metastable clusters. To estimate the metastability of assembly, we define the lifetime of a cluster *τ*, i.e., the timespan that a set of interacting particles remains assembled. We assign long‐lived metastability to clusters with *τ* larger than the critical lifetime *τ*_c_ = 100 × *τ*_R_ ≈ 7 s where *τ*_R_ is the average time required for an active particle to move one particle radius. Such enduring motile assemblies emerge in contrast with short‐lived clusters that form and break with *τ* < *τ*_c_. We measure the probability distribution of the normalized cluster time *τ*/*τ*_c_ for pairs of passive particles, and pairs of passive and active particles, interacting in the AC electric field (Figure 3B). For each class of pair, we extract *τ* from 30 distinct pairs to determine their metastability. Each observed passive–passive pair assembled into long‐lived metastable clusters with *τ*/*τ*_c_ >> 1. Conversely, each observed passive‐linearly propelled pair interacted for a very short time with a mean *τ*/*τ*_c_ of ≈0.05 (<< 1). Interactions between passive and helically propelled particles display a bimodal distribution of *τ*/*τ*_c_ with ≈10% probability of forming short‐lived metastable clusters that disassemble shortly after collision. The remaining ≈90% of observed passive‐helically propelled interactions gave rise to clusters that were rendered motile by the active component.

The origin of finite cluster lifetimes in the active‐passive particle mixtures is the combination of field‐induced dipolar and fluid‐mediated hydrodynamic interactions leading to the observed non‐reciprocity. The electric field distribution and the resulting fluid flows around a patchy particle in an AC electric field are strongly influenced by the morphology of the metal patch. Using 2D finite element modeling in COMSOL Multiphysics, we solve the coupled electric field and electroosmotic flows around a patchy particle as a function of the fraction *f* of the particle surface covered by the thin gold patch (for details, see Note S5, Supporting Information). Our calculations reveal that the electric field magnitude is largest at the poles, regardless of the area of the thin patch (Figure 3C,D). However, the electroosmotic flows are highly localized at the interface between the gold patch and the polystyrene surface, and changing the patch morphology significantly alters the local velocity field. For Janus particles with a large patch fraction (*f* = 0.5), the strongest ICEO flows occur at the poles, which inhibits assembly with passive particles. In contrast, reducing the patch fraction (i.e. *f* < 0.5) shifts the ICEO flows away from the poles and lowers the hydrodynamic barrier to dipolar assembly (Figure 3E–G). While the 2D model does not capture the non‐linear motion of the particles, the observed shift in ICEO flows from polar to equatorial regions and the corresponding impact on interparticle interactions remains valid. These findings highlight the critical role of patch fraction in the co‐assembly of patchy and non‐patchy particles, leading to the formation of metastable states as observed in our experiments. Moreover, they highlight the impact of non‐reciprocal interactions in forming metastable active assemblies.

## 4 Structure, Dynamics, and Reconfigurability of Active–Passive Clusters

**Figure 4:**
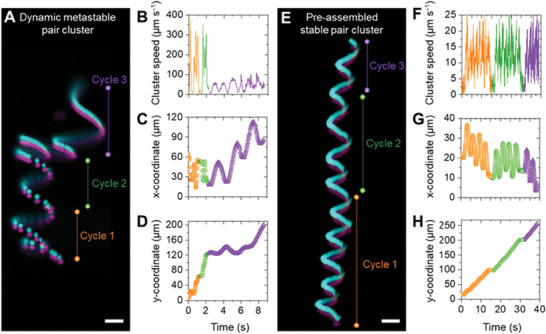
Dynamics of metastable and pre‐assembled stable pair clusters. A) Overlay of fluorescence microscopy images recording the motion of a dynamic metastable pair cluster as the external AC electric field is toggled on and off for 3 cycles. The cluster is assembled by the field and disassembles in its absence. B) The speed of the active cluster differs as the field is toggled on and off, and its trajectory varies in (C) the x and (D) y dimensions upon each disassembly/assembly cycle. E) Overlay of fluorescence microscopy images recording the motion of a pre‐assembled stable pair cluster as the field is toggled on and off for three cycles. The cluster is pre‐assembled due to stronger van der Waals interactions and remains assembled irrespective of the presence or absence of the external electric field. The role of the electric field is to drive alignment and motion of the pre‐assembled doublet cluster. F) The cluster speed is ≈12 µm s^−1^ when the field is on and remains equal for each cycle. More importantly, the trajectory of the particle is nearly identical in all three cycles, as observed by tracking cartesian coordinates of the center of mass of the clusters shown in (G) x and (H) y. The orange, green, and purple colors in the overlay images and plots represent the three different cycles (1–3) of the field on/off experiment. The applied field strength in all experiments shown above is 300 V cm^−1^ at 10 kHz. Scale bars in (A) and (E) are 20 µm.

Motile metastable clusters form due to a combination of a bulk reciprocal dipolar attraction and a fluid‐mediated non‐reciprocal interaction from the localized ICEO flows. The reciprocal component of the net interaction promotes the formation of stable structures. The non‐reciprocal component introduces reconfiguration by promoting the spontaneous reorganization of particles within a cluster, thus enabling a versatile transition between multiple metastable states. The isolated metastable clusters formed by superposition of reciprocal and non‐reciprocal interactions display motility and reconfiguration which depends on their time‐varying morphology, i.e., the dynamics of motion vary as the cluster reconfigures. This dynamic interaction energy landscape implies that the configuration of a helically propelled and a passive particle assembled in the AC electric field varies each time the field is toggled on and off. We demonstrate this by tracking the motion of two types of doublet clusters as we cycle the external field on and off. The first type represents dynamic metastable doublets which are assembled in situ via dipolar forces, and the second type represents pre‐assembled stable doublets which are permanently linked via van der Waals forces. Dynamic metastable doublets form when the electric field is introduced, move in helical trajectory, and disassemble when the field is removed (**Figure** **4**A,B). As the field is toggled on and off, the trajectory of the isolated dynamic cluster in each cycle is never the same, as either the helix pitch or diameter or both differ (Figure 4C,D; Figures S5–S6 and Video S3, Supporting Information). Conversely, the motion of pre‐assembled stable doublets maintains similar features as the electric field is cycled on and off (Figure 4E–H; Figures S5 and S7, and Video S3, Supporting Information). Note that the pre‐assembled clusters are synthesized by drying a mixture of active and passive particles on a glass substrate followed by gentle scraping and resuspension in deionized water. While these clusters could display a variety of trajectories in AC electric field depending on the symmetry of the formed cluster, the dynamics remain similar for every field on/off cycle, highlighting their stable nature. The variation in cluster symmetry and consequent trajectory for a dynamic doublet provides evidence that, at identical electric fields, the interaction between active and passive particles forms transient metastable configurations. In purely active systems, this stochastic reconfiguration from non‐reciprocal interactions leads to continuous reconfiguration between short‐lived states. By overlapping this with bulk attraction, we obtain clusters with long‐lived metastability that reconfigure via interaction with other clusters or the environment yet maintain a core structure.

**Figure 5:**
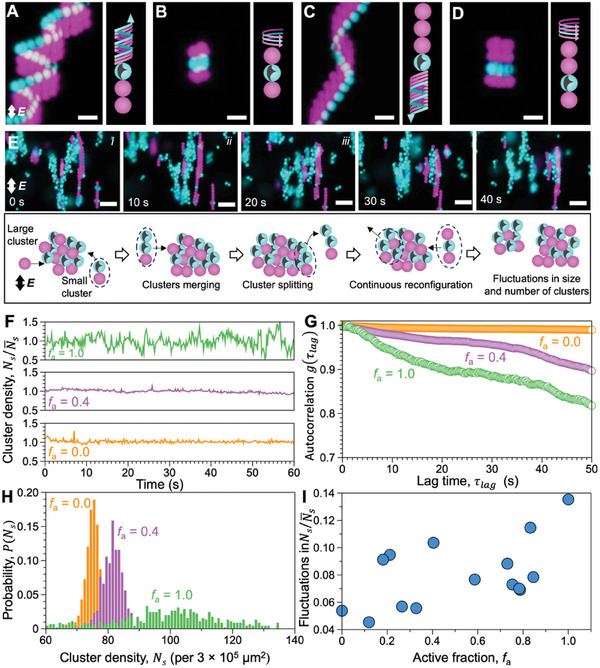
The motion of isolated clusters and fluctuations in interacting clusters. A–D) Overlays of fluorescence microscopy images showing the (A,C) helical motion of isolated triplets and quadruplets with the active particle at either end of the assembly. B,D) Active particles sandwiched by two passive particles rotate in place, without significant translation. This occurs due to disruptions to the ICEO flows caused by the passive particles. Scale bars in (A–D): 10 µm. E) Fluorescence microscopy images (top) and schematics (bottom) displaying the merging and splitting of interacting clusters. Scale bars in (E): 20 µm. F) Cluster number density *N_s_* (number per 3 × 10^5^ µm^2^) normalized by the time‐averaged cluster number density ${\bar{N}}_{s}$. G) Autocorrelation function of the cluster number density for increasing the fraction of active particles present in the active‐passive mixtures i.e., *f_a_*. The characteristic decay of *g*(τ_*lag*_) with τ_*lag*_ occurs at shorter timescales upon increasing *f_a_*. H) The probability distribution function *P*(*N_s_*) of the cluster number density for *f_a_* = 0, 0.4, and 1.0. The mean and standard deviation of the *P*(*N_s_*) increases with an increase in *f_a_*, thus indicating greater fluctuations and structural reconfiguration occurring upon increasing the relative population of the active particles in the mixture. I) The change in fluctuations in the number density of clusters quantified using standard deviation of the cluster probability distribution as shown by three representative cases in (H).

The patchy particles are the active “engines” of the metastable assemblies and are responsible for the observed cluster dynamics. The translation and rotation of these active particles in the AC electric field are modulated by the assembly as the transient morphology of the isolated clusters determines the ICEO flows. This gives rise to dynamic characteristics to the dispersion which we observe via experiments at low particle concentrations. The low particle counts favor the assembly of long‐lived isolated small clusters that form typical chain‐like structures and are likely to have a single active component. When the isolated active particle assembles at either end of the chain, it simultaneously rotates and translates, leading the cluster through helical trajectories with the engine particle always facing forward (**Figure** **5**A,C; Figure S8 and Videos S4 and S5, Supporting Information). The linear and rotational velocity of these isolated clusters follow the typical ICEP kinematics, i.e., they scale linearly with the square of the electric field strength (Figure S8, Supporting Information). When the active particle is sandwiched between multiple passive particles, the translational ICEP flows are restrained and only the rotational dynamics are retained (Figure 5B,D and Video S5, Supporting Information). Increasing particle concentrations promotes the interactions between previously isolated clusters. The interacting clusters have a marked tendency to reconfigure via spontaneous splitting and merging in proximity of their active components (Video S6, Supporting Information), highlighting a unique role that active particles play when mixed with passive particles.

The non‐reciprocal interactions between active and passive particles and the resultant metastability of the assembled clusters underlie their shape‐morphing behavior in concentrated dispersion where individual clusters begin to interact. This is further evidenced with increasing particle concentrations giving rise to dynamically reconfiguring motile clusters of tens or hundreds of particles (Video S6, Supporting Information). The interacting active clusters display more complex dynamics than what is observed in isolated clusters. This is due to the combinatorial effects of dipolar and ICEP forces, not only within a cluster but also between neighboring clusters. The presence of active particles in dense suspension of the passive particles induces continuous reconfiguration of clusters which, in their absence, are known to form long stable bundles and crystalline structures.^[^ ^56^, ^57^ ^]^ As large clusters move and interact, they split and merge via scission and agglomeration of smaller clusters (Figure 5E and Video S6, Supporting Information). We identify the effect of the fraction of active particles in the mixture, *f*_a_, on the structural reconfiguration. For each experiment, we allow clusters to form and reconfigure for 60 s to reach a steady state. In this state, we measure the temporal evolution of the number density of clusters *N*_s_ as they split and merge and in the size of clusters as the reconfiguration takes place over time. The temporal fluctuations in *N_s_* \${\bar{N}}_{s}$ increase with increasing *f_a_* as shown in Figure 5F for dispersions with only passive particles (*f_a_* = 0.0), only active particles (*f_a_* = 1.0) and an intermediate mixture (*f_a_* = 0.4). Assuming the temporal fluctuations in *N*_s_ to be similar to the ones generated in the intensity of scattered light from a dispersion, we can qualitatively compare the reconfiguration timescales in these active‐passive mixtures via the autocorrelation function *g*(τ_*lag*_), defined as^[^ ^58^ ^]^

(1) $$g\left(\tau_{lag}\right)=\frac{\langle N_{s}\left(t\right)N_{s}\left(t+\tau \right)\rangle }{{\langle N_{s}\left(t\right)\rangle }^{2}}$$

where τ_*lag*_ is the lag time, and 〈 · 〉 represents the expected value of *N_s_*. In our experiments, we find that *g*(τ_*lag*_) remains nearly 1 for long lag times (tens of seconds) when *f_a_* = 0.0, indicating lack of any significant fluctuation. The characteristic decay of the *g*(τ*_lag_*) with τ_*lag*_ occurs at shorter timescale upon increasing the fraction of active particles present in the mixture (Figure 5G). This suggests that replacing “reciprocal interparticle links” with “non‐reciprocal links” enhances fluctuations and promotes structural reconfiguration in mixtures of active‐passive particles. It is important to note that exact timescales of structural reconfiguration cannot be precisely extracted from *g*(τ_*lag*_), as the decay rates themselves vary over time.^[^ ^59^ ^]^ Regardless, *g*(τ_*lag*_) provides a qualitative insight into the dependence of structural reconfiguration on *f_a_* ​. A similar relationship between structural reconfiguration and non‐reciprocity in colloidal assemblies has been demonstrated recently in simulations by Klapp and co‐workers.^[^ ^60^ ^]^

Increasing *f*_a_ increases the number of clusters involved in reconfiguration, as more active particles become involved in transferring between clusters and shepherding passive particles also leading to higher fluctuations (Figure 5F,G). This is further evidenced by the increase in the mean and standard deviation of probability distribution function of the cluster number density (*P*(*N_s_*)) with increasing *f_a_* (Figure 5H). Correspondingly, the degree of fluctuations in the dispersion quantified by the standard deviation of the *P*(*N_s_*) increases with increasing *f_a_* (Figure 5I). In addition, the size *c* of these clusters involved in reconfiguration increases with *f*_a_, augmenting the fluctuations in average cluster size (Figure S9, Supporting Information). These observed dynamic structures somewhat resemble those previously shown in simulations of interacting active‐passive particles.^[^ ^61^, ^62^ ^]^ The fraction of motile particles controls the frequency and intensity of fluctuations in active clusters.^[^ ^63^, ^64^ ^]^ This highlights how the overlap of bulk reciprocal and fluid‐mediated non‐reciprocal interactions can be tuned to either promote or discourage stable assembly in otherwise fully phase‐separating mixtures.

## 5 Conclusion

Shedding light onto the elusive overlap of reciprocal and non‐reciprocal interactions reveals a rich landscape for the assembly of metastable microstructures. These display the out‐of‐equilibrium dynamics typical of systems of active particles but combine it with the programmable structuring traditionally associated with field‐driven assembly of passive particles. The resulting colloidal matter emerges from the interplay of equilibrium and non‐equilibrium forces respectively with constant bulk and transient fluid‐mediated interactions. The design of patch shape impacts this interplay and provides a method for controlling the degree of reciprocity in the net interaction between active and passive particles. Helically propelling particles with chiral triangular patches interact with passive particles in crowded environments forming isolated motile structures with long‐lived metastability. This degree of reciprocity is tuned by the fraction of active components in the mixture, demonstrating further control over the collective organization of colloids far‐from‐equilibrium. The increased understanding of the mechanisms of non‐reciprocal assembly can exploit this common behavior in active matter to harness micro‐ and nanoparticle organization. Our study bridges two existing research currents in the field of active matter: the exquisite control over single‐particle dynamics and the random organization of collectives via understanding of the relevant steady and transient forces. The particle assembly and cluster growth/fragmentation are unavoidably coupled to hydrodynamics of particles and clusters,^[^ ^65^ ^]^ including separation‐ and shape‐dependent hydrodynamic forces. Further studies on coupling to the transient nature of hydrodynamics would provide a basis toward predictive understanding of unprecedented microstructures from non‐reciprocal assembly under far‐from‐equilibrium conditions.

## 6 Experimental Section

### Particle Fabrication and Characterization

Two aqueous suspensions of fluorescent polystyrene microspheres (*R* = 2.6 µm) were purchased from Magsphere Inc. and were washed with deionized water via three cycles of centrifugation to remove excess surfactant (Figure S10, Supporting Information). One set of particles were labeled with fluorescein isothiocyanate dye and represented in magenta, and the other set was labeled with Nile Red dye and represented in cyan, for visual accessibility. The particles were highly charged and colloidally stable in deionized water with zeta potentials of ≈−70 mV for the magenta and −55 mV for cyan particles, as measured using the Anton Paar Litesizer 500. To fabricate patchy particles, each suspension was concentrated to ≈20 wt.% before depositing the particles on a glass substrate as monolayers using the convective assembly method. Metal vapor deposition was done in a Thermionics Laboratory VE‐90 thermal evaporator, with a deposition rate of 0.5 and 0.1 nm s^−1^ respectively for a 10 nm Cr layer followed by a 30 nm Au layer. The method of glancing angle vapor deposition is used to deposit chiral triangular patches.^[^ ^66^ ^]^ This involves varying the angle of incidence between the particle monolayer and the metal vapor to tune the shape of the deposited patch due to self‐shading among microspheres (Figure S11, Supporting Information). The patchy particles were transferred from the glass slide into a vial using a spatula and re‐dispersed in deionized water by sonication before each experiment.

### AC Electric Field Setup

The coplanar electrodes used to generate the AC electric field were fabricated via metal vapor deposition of 10 nm Cr layer followed by 100 nm Au layer onto glass slides with a 2 mm wide mask. All glass slides used were presoaked in NoChromix solution for 12 h, washed with DI water, and dried in a convection oven before metal vapor deposition. A 100 µm thick Teflon spacer was used to fabricate 2 × 2 mm square chamber encompassing the gap between the electrodes. In a typical experiment, the particle suspension was micro‐pipetted into the gap between the electrodes and sandwiched between the glass slide and a cover glass in a Hele‐Shaw cell type of configuration. To minimize the influence of sedimentation and substrate‐related hydrodynamic interactions, all measurements were conducted within 5–10 min after the sample preparation, and force measurements were performed only on particles positioned at a minimum distance of ≈20 µm from the substrate.

The coplanar electrodes were then connected to a function generator (Agilent) and a high‐voltage amplifier (Tegam) to apply a square‐wave AC electric field of magnitudes up to 700 V cm^−1^ and 10 kHz frequency monitored by an oscilloscope (Keysight).

### Microscopy

The particle dynamics were observed and recorded using a Leica DM6 upright microscope. High‐speed imaging in the brightfield mode was used to capture interparticle collision events with framerates between 100 and 300 fps using the Leica DFC9000 GTC camera. All other imaging was done in fluorescence mode using the EL 6000 light source through the EGFP/mCherry filter cube (Chroma) for simultaneous imaging of green (magenta) and red (cyan) fluorescence with the MTR3 CMOS color camera.

### Image Analysis

Microscopy data was processed by particle tracking using the Trackmate plugin^[^ ^52^ ^]^ within the ImageJ software.^[^ ^53^ ^]^ This process yielded position coordinates in the 2D imaging plane for all recorded interactions. For analyzing helically‐propelling particles approaching passive particles, interactions were tracked only when the motion of the particles was in the imaging plane before collision. Position coordinates for each particle and the associated timestamps were input to a MATLAB script for improving signal‐to‐noise ratio. Forces were extracted from these coordinates while minimizing error propagation due to differentiation by using the method of low‐bandpass filtering within the signal processing toolbox in MATLAB (Note S3, Supporting Information). The data on cluster reconfiguration was extracted by identifying areas corresponding to clusters from binarized recordings. *N*_s_ was determined by filtering the cluster count to areas equivalent to at least two particles and smaller than the pre‐computed average cluster size. The cluster size *c* was tracked for individual large clusters as they split and agglomerated smaller clusters.

## Conflict of Interest

The authors declare no conflict of interest.

## Supporting information



Supporting Information

Supplemental Video 1

Supplemental Video 2

Supplemental Video 3

Supplemental Video 4

Supplemental Video 5

Supplemental Video 6

## Data Availability

The data that support the findings of this study are available from the corresponding author upon reasonable request.
