# Supplementary material for: Non‐Reciprocity, Metastability, and Dynamic Reconfiguration in Co‐Assembly of Active and Passive Particles
Source: Adv Sci (Weinh). 2024 Dec 4;12(4):2409489. doi: 10.1002/advs.202409489 (PMC11775524; doi:10.1002/advs.202409489)
Supplement: Supplementary file 1 — Supporting Information [file ADVS-12-2409489-s007.pdf]

ADVANCED SCIENCE
Open Access

Supporting Information

for *Adv. Sci.*, DOI 10.1002/advs.202409489

Non-Reciprocity, Metastability, and Dynamic Reconfiguration in Co-Assembly of Active and Passive Particles

Ahmed Al Harraq, Ruchi Patel, Jin Gyun Lee, Ope Owoyele, Jaehun Chun and Bhuvnesh Bharti**

Supporting Information

Non-reciprocity, metastability, and dynamic reconfiguration in co-assembly of active and passive particles

Ahmed Al Harraq*, Ruchi Patel, Jin Gyun Lee, Ope Owoyele, Jaehun Chun, and Bhuvnesh Bharti*

A. Al Harraq, R. Patel, J. G. Lee, B. Bharti
Cain Department of Chemical Engineering, Louisiana State University, Baton Rouge, LA 70803, USA
Email: bbharti@lsu.edu

A. Al Harraq
Center for the Physics of Biological Function, Princeton University, Princeton, NJ 08544, USA
Email: alharraq@princeton.edu

J. G. Lee
Department of Chemical and Biological Engineering, University of Colorado, Boulder, CO 80303, USA

O. Owoyele
Department of Mechanical and Industrial Engineering, Louisiana State University, Baton Rouge, LA 70803, USA

J. Chun
Physical and Computational Sciences Directorate, Pacific Northwest National Laboratory, Richland, WA 99354, USA

Supporting Note S1

Colloidal dipole-dipole interactions

The polarization of a microsphere in an ac electric field drives a dipole-dipole interaction which is respectively attractive and repulsive along the parallel and orthogonal to the direction of electric field strength $\boldsymbol{E}$. The interaction energy ($\boldsymbol{U}$) between two polarized particles follows from point-dipole approximation as[1]

$$\boldsymbol{U} = \frac{\boldsymbol{\mu}^2}{d^3}[1 - 3\cos(\theta)]$$

Where d is the distance between the pair of particles, and θ is the angle between the vector connecting the particles and the direction of the applied field $\boldsymbol{E}$. The dipole moment $\boldsymbol{\mu}$ is given as

$$\boldsymbol{\mu} = 4\pi\, \varepsilon_{\mathrm{m}}\, K_{\mathrm{CM}}\, R^3 \boldsymbol{E}$$

Where R is the radius of the particle, $K_{\mathrm{CM}} = (\varepsilon_{\mathrm{p}} - \varepsilon_{\mathrm{m}})/(\varepsilon_{\mathrm{p}} + 2\varepsilon_{\mathrm{m}})$ is the real part of the Clausius-Mossotti function, ε_m and ε_p are the complex permittivities of the suspending medium and particle with the counterion double layer, respectively. Pole-to-pole attraction reliably leads to the assembly of two interacting isotropic microspheres and is commonly employed for the fabrication of chain-like structures of passive particles (Note Figure S1)

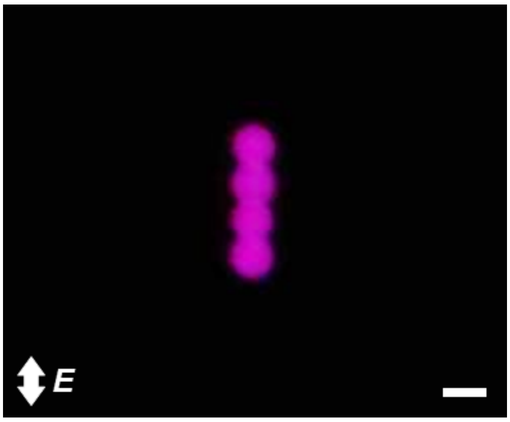

Note S1 Figure S1. Polarized passive particles assemble into passive chain-like cluster in ac electric field of 300 V cm^{-1} strength and 10 kHz frequency. Scale bar: 5 µm.

Supporting Note S2

Induced-charge electrophoresis

Dielectric particles with a metal patch have different polarizability in the dielectric and metal surfaces. Such asymmetry in polarization under ac electric fields results in stronger electroosmotic slip on the metal side than the non-metal side. This causes the phenomenon termed induced-charge electrophoresis (ICEP) theorized by Squires and Bazant[2] and originally observed in the Velev group using metallodielectric Janus particles.[3] Polymeric particles with a gold coated hemisphere self-propel perpendicular to the externally applied ac electric field direction and with the polymer hemisphere facing the direction of motion (as shown in Figure 1 and Video S1, Supporting Information). The linear ($\boldsymbol{V}_{\mathrm{ICEP}}$) and angular velocity ($\boldsymbol{\Omega}_{\mathrm{ICEP}}$) of a particle moving via ICEP is given as[2]

$$\boldsymbol{V}_{\mathrm{ICEP}} = -\frac{\oint_{\mathrm{S}} \boldsymbol{u}_{\mathrm{s}}(\mathrm{r})\mathrm{dr}}{\oint_{\mathrm{S}} \mathrm{dr}}$$

$$\boldsymbol{\Omega}_{\mathrm{ICEP}} = -\frac{\oint_{\mathrm{S}}[\boldsymbol{r} \times \boldsymbol{u}_{\mathrm{s}}(\mathrm{r})]\mathrm{dr}}{2\oint_{\mathrm{V}} \mathrm{dr}}$$

where $\boldsymbol{u}_{\mathrm{s}}(\mathrm{r})$ is the slip velocity distribution on the surface of a particle (including metal patch) and $\boldsymbol{r}$ is the unit normal vector. In the case of a Janus sphere, $\boldsymbol{\Omega}_{\mathrm{ICEP}} = 0$ as $\oint_{\mathrm{hemi-sph}}[\boldsymbol{r} \times u_{\mathrm{s}}(\mathrm{r})]\mathrm{dr} = 0$ because the integral of an odd function over a symmetric interval (i.e., over the hemispherical patch) is zero. In addition, $\boldsymbol{V}_{\mathrm{ICEP}}$ reduces to $\boldsymbol{V}_{\mathrm{ICEP}} = -\frac{9\varepsilon_{\mathrm{m}}R\boldsymbol{E}^2}{64\eta(1+\delta')}$, where δ' is the ratio of capacitances of the compact and diffuse double layer on the particle and η is the viscosity of the surrounding fluid. We recently showed how equipping particles with chiral triangular patches breaks the mirror symmetry of the Janus configuration, introducing a rotational element to the ICEP motion[4]. Combining translational and rotational components lead to helical self-propulsion as shown in Figure 1 and Video S1, Supporting Information.

Supporting Note S3

Finite Impulse Response (FIR) filtering for extracting interaction velocities

In our analysis, velocity of the particle is calculated by taking the time derivative of the position vectors in space extracted from videos recorded at high frame rate using TrackMate plugin[5] on ImageJ software.[6] In our experiments, the time period of Brownian fluctuations is less than 0.1 s, and that of the particle collision event is about 1 s. (Note S3 Figure S1). Thus, the high frequency Brownian fluctuations (start of the event) and velocity changes due to millisecond scale interactions are decoupled in frequency domain by an order of magnitude.

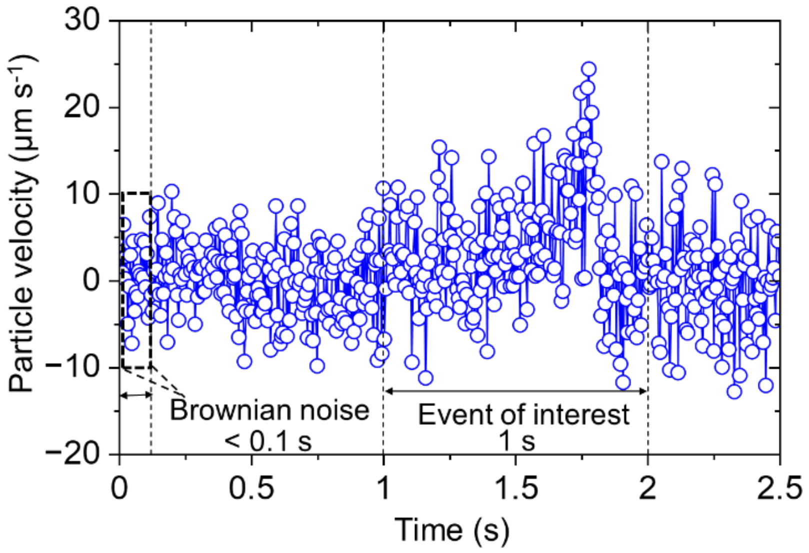

Note S3 Figure 1. Particle velocity extracted by calculating the time derivative of the position vectors. The rectangle highlights the high frequency Brownian noise acquired at the start of the event which persists for < 0.1 s. The region from time 1 s to 2 s represents the low frequency event of interest during which the interparticle interaction occurs.

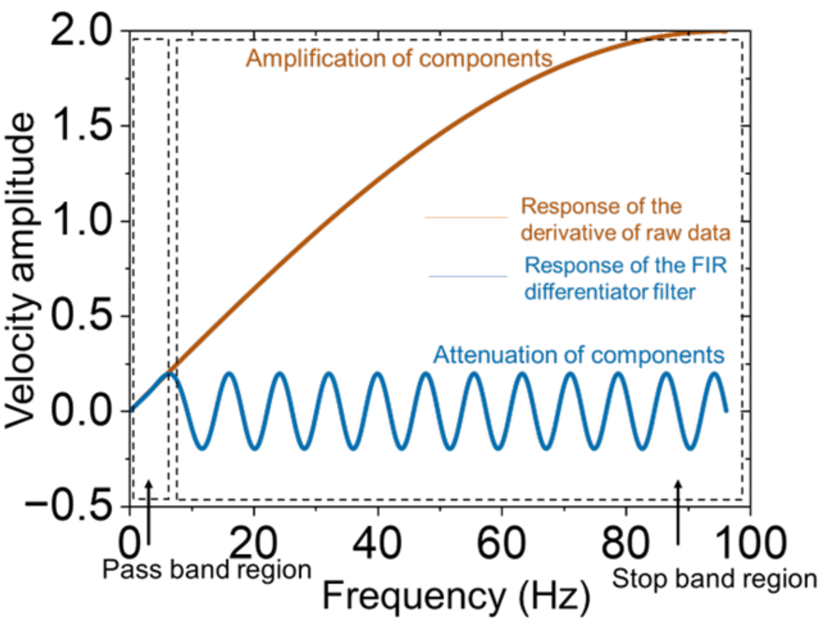

Note S3 Figure S2. Zero phase response plot of the FIR filter representing scaled velocity amplitude as a function of frequency. The orange curve represents the velocity amplitude, obtained by taking the time derivative of the position data and normalized from 0 to 2. The blue curve represents the corresponding response of the FIR differentiator filter. The response of the FIR filter is equal to that obtained by differentiating the unfiltered data, in the pass band region. The response of the FIR filter is highly attenuated, while that of the differentiated data is highly amplified, in the stop band region.

A 50th order FIR differentiator filter is designed to extract the signal by attenuating the high frequency Brownian noise.[7,8] The zero-phase response plot represents the scaled amplitude (normalized from 0 to 2) of interaction velocities in frequency domain. The response of the velocities extracted by taking the time derivative of raw position vector data and that using FIR differentiator filter is shown in Note S3 Figure S2.

In the pass band region, the response to the FIR differentiator filter is same as that obtained by differentiating the unfiltered data. This implies that the filtered signal is coupled to the Brownian noise in this frequency domain. In the stop-band region, the unfiltered components (signal + noise) are amplified on differentiation, whereas they are attenuated by the FIR differentiator filter. This feature of the filter plays a role in decoupling the Brownian noise from the information in the signal and helps in extraction of velocity changes due to interactions.

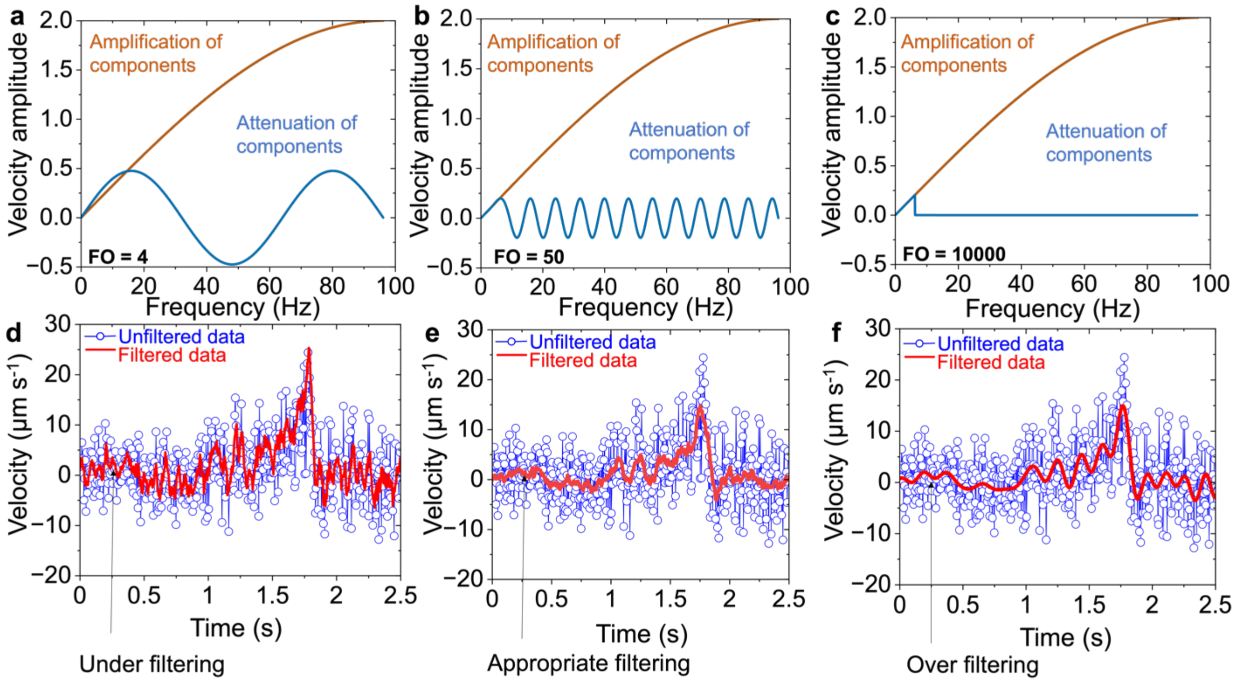

Note S3 Figure S3. Selection of an optimum filter order, FO to extract signal, after maximum attenuation of Brownian noise. (a, b, c) The orange curve represents the velocity amplitude, obtained by taking the time derivative of the unfiltered position data and normalized from 0 to 2. The blue curve represents the corresponding response of the FIR differentiator filter. (d,e,f) The blue circles represent the velocity data obtained by taking the time derivative of the unfiltered position vectors. The red curve represents the filtered velocities extracted using the FIR differentiator filter. (a) A low order filter, FO = 4 displaying similar responses on differentiation of unfiltered and the FIR differentiator filtered data up to high frequencies. (d) Red curves fluctuate like the blue symbols, indicating the presence of Brownian noise in filtered signal. (b) An appropriate order filter, FO = 50 displaying similar responses to the unfiltered data at low frequencies but attenuating the noise at high frequencies (e) Red curve moderately varies as blue symbols, indicating the decoupling of high frequency Brownian noise from the low frequency event of interest. (c) A high order filter, FO = 10000 displaying different responses to differentiation of unfiltered and filtered data at most frequencies. (f) Red curve barely overlaps with the blue symbols displaying attenuation of signal accompanied with noise up to extremely high frequencies, leading to loss of signal.

Filter order, FO: An appropriate filter order is essential to extract changes in interaction velocities, with maximum attenuation of Brownian noise. A filter of low order gives a similar response to the differentiation of unfiltered and FIR differentiator filtered data up to high frequencies. This is indicative of the presence of Brownian noise in the extracted interaction

velocity signal (Note S3 Figure S3A, D). A filter of high order attenuates components significantly even at low frequencies. This is indicative of the attenuation of the signal along with the Brownian noise, leading to loss of significant data points in the event of interest (Note S3 Figure S3C, F). A filter of an appropriate order attenuates noise at high frequencies while it gives response like differentiation of unfiltered data at low frequencies. Thus, a filter of this design is appropriate to decouple the high frequency Brownian noise from the low frequency event of interest which occurs on a much larger time-period as compared to Brownian diffusion (Note S3 Figure S3B, E).

Supporting Note S4

Unsteady Stokes flow of particles in ac electric field

An ac electric field of frequency ω drives the polarization of the electrical double layer (EDL) of the particle which follows the polarity of the alternating field until $1/\omega$ is comparable to the ion relaxation time (τ_D) in the fluid. In our experiments, $1/\omega \sim 10^{-4}$, and $\tau_D \sim 10^{-7}$, i.e., $\omega \ll 1/\tau_D$, therefore, the ions in the vicinity of the particle will oscillate with a frequency $\sim\omega$ with an amplitude of the order of the size of the colloidal particle. Therefore, the ac electric field causes an oscillating transient motion of the EDL and subsequent acceleration of the surrounding fluid. The resulting particle dynamics are described by an *unsteady* Stokes flow[9,10] such that F_d is time-dependent, opposed to a temporally invariant Stokes drag force that is a typical case, and includes additional forces associated with

i) the transient development of the momentum boundary layer next to the oscillating particle involving the history of the motion, called the Basset force

ii) an added mass effect from an extra force or resistance to overcome the inertia of the surrounding fluid.

Such oscillatory motions cause diffusion of vorticity through the fluid over a length δ, scaled as $(\nu/\omega)^{1/2}$ where ν is the kinematic viscosity of the fluid. The importance of the transient nature of the flow is determined by the dimensionless term $\lambda = R/\delta = R(\omega/\nu)^{1/2}$ where R is the particle radius. By using the transient Oseen tensor along with the singularity method, Kim and Karrila demonstrated that the $O(\lambda)$ and $O(\lambda^2)$ correction terms correspond to the contributions from the Basset forces and added mass to the time-dependent drag force on the particle, in addition to the quasi-steady Stokes drag from the $O(1)$ term[9]. In our case, we have a small but finite λ (about 0.1), indicating that the particle velocity varies over time. Note that, in our case, δ is comparable to the amplitude of the oscillation, i.e., $O(R)$ but $R^2\omega/\nu \ll O(1)$, highlighting that the time-dependent component of fluid acceleration (i.e., $\partial v/\partial t$ term in Navier Stokes equation) is still maintained but the inertial component (i.e., $v \cdot \nabla v$ term in Navier Stokes equation) is still negligible.

Supporting Note S5

Finite element analysis

The electric field distribution and the corresponding fluid flows around a patchy particle in an ac electric field are affected by the size of the metal patch. We use finite element modeling using the COMSOL Multiphysics software package to identify changes in the coupled electric field and electroosmotic flows around a particle as a function of patch fraction *f*. This is defined as the fraction of particle surface that is covered by the single metal cap. We perform the model calculation on a circular particle of radius 2.6 µm surrounded by water in a 2D simulation box. We added a 30 nm shell occupying the variable fraction *f* of the particle surface and represented the electrical double layer as a 30 nm shell present selectively on the remaining non-patchy surface. This assumption prevents potential artifacts of an asymmetric geometry of the particle within the box. We calculate the electric field distribution using the electric current module to solve Maxwell's equations. This solution was coupled with the creeping flow module to obtain the corresponding fluid velocity distribution. Details of the numerical setup are shown in Note S5 Figure S1.

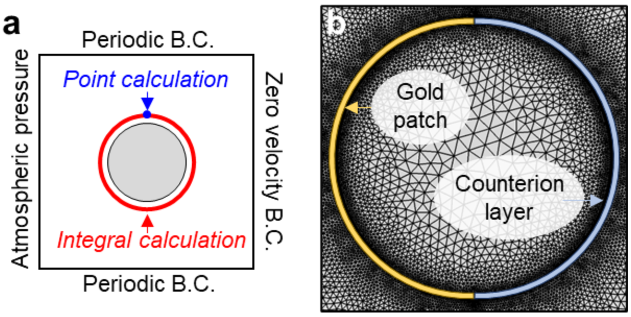

Note S5 Figure S1. Details of the numerical setup for finite element analysis. (a) Schematic of 2D simulation box built within COMSOL Multiphysics with corresponding boundary conditions. Integral values around the surface of the particle (red line) and point values at the pole (blue dot) were extracted from the simulations. (b) Snapshot of the triangular mesh applied to the simulation with overlay showing the thin gold patch (yellow) and counterion layer (light blue).

We perform the calculation of electric field and electroosmotic velocity distribution around a particle with increasing patch size from $f = 0$ to 0.5. In addition, we compute point and integral values of electric field and velocity obtained respectively from a point 100 nm away from the pole and by integrating for all such points around the surface. For all *f*, the electric field strength remains highest in the polar regions and lowest in the equatorial regions, highlighting the polarization of the particles in the direction of applied field (Note S5 Figure S2A).

Supporting Figures

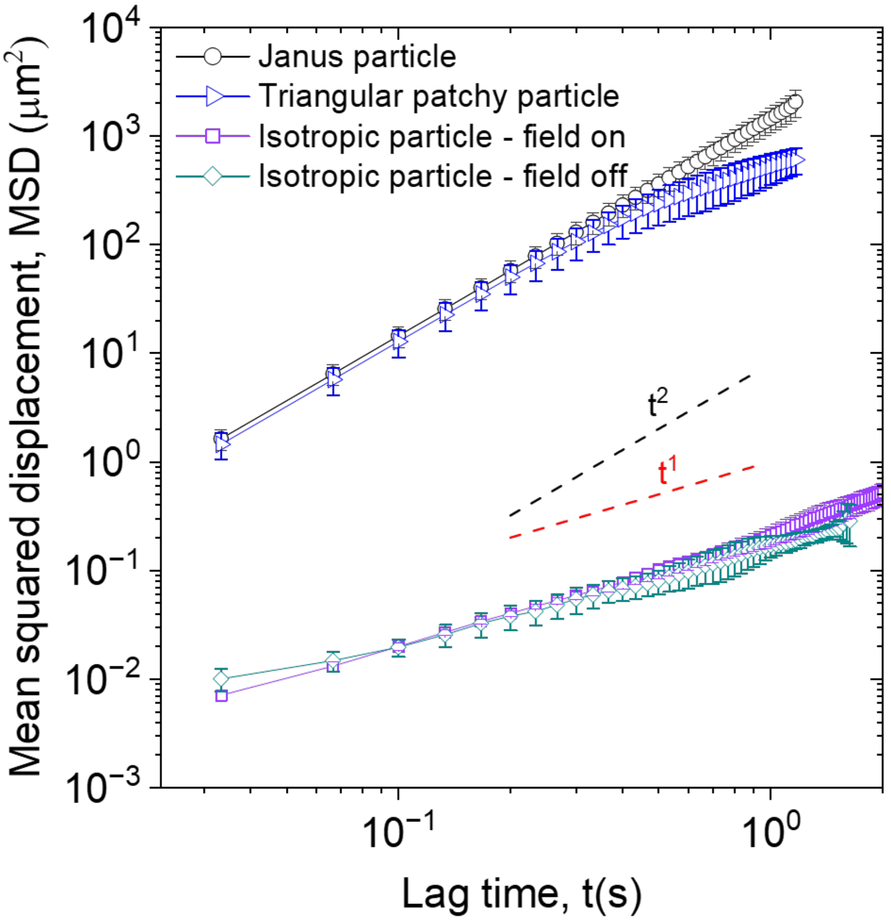

Figure S1. Mean squared displacement (MSD) vs lag time extracted for a Janus particle, triangular patchy particle, isotropic particle with field off, and isotropic particle with field on. The experimental MSD data points of linearly propelled Janus particle (gray circles) and helically propelled triangular patchy particle (blue triangles) scale with t^2 as shown by the dashed black line. The experimental MSD data points of isotropic particle in field (purple squares) and isotropic particle without field (cyan rhombus) scale with t as shown by dashed red line.

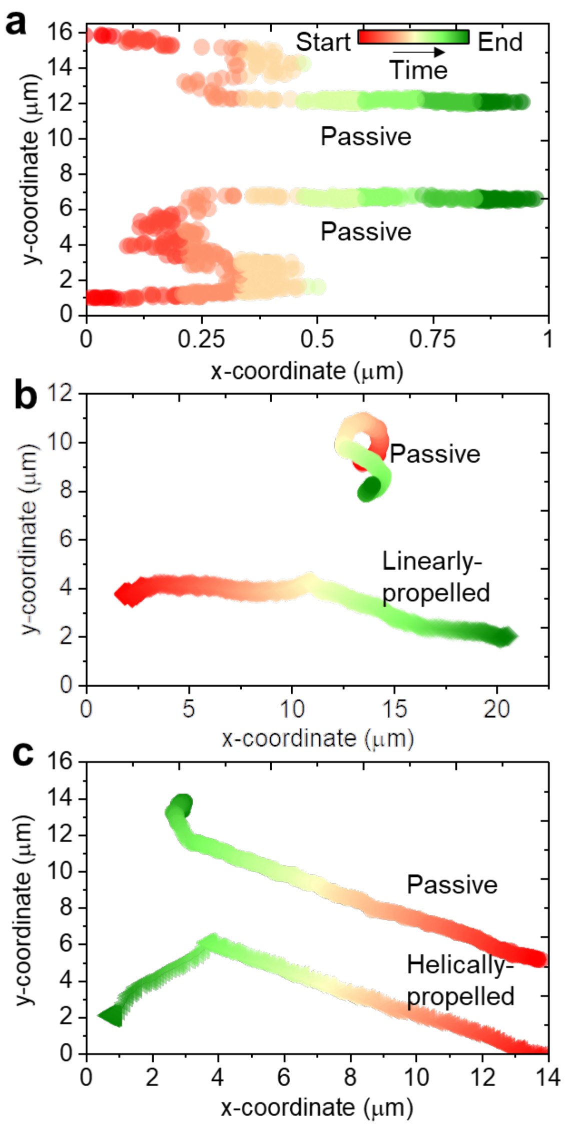

Figure S2. Tracked coordinates of interacting particles in a global reference frame. The coordinates obtained using the Trackmate plugin reflect the motion of a passive particle with (a) another passive particle with which it assembles, (b) a linearly-propelled particle which it deflects, and (c) helically propelled particles which it assembles.

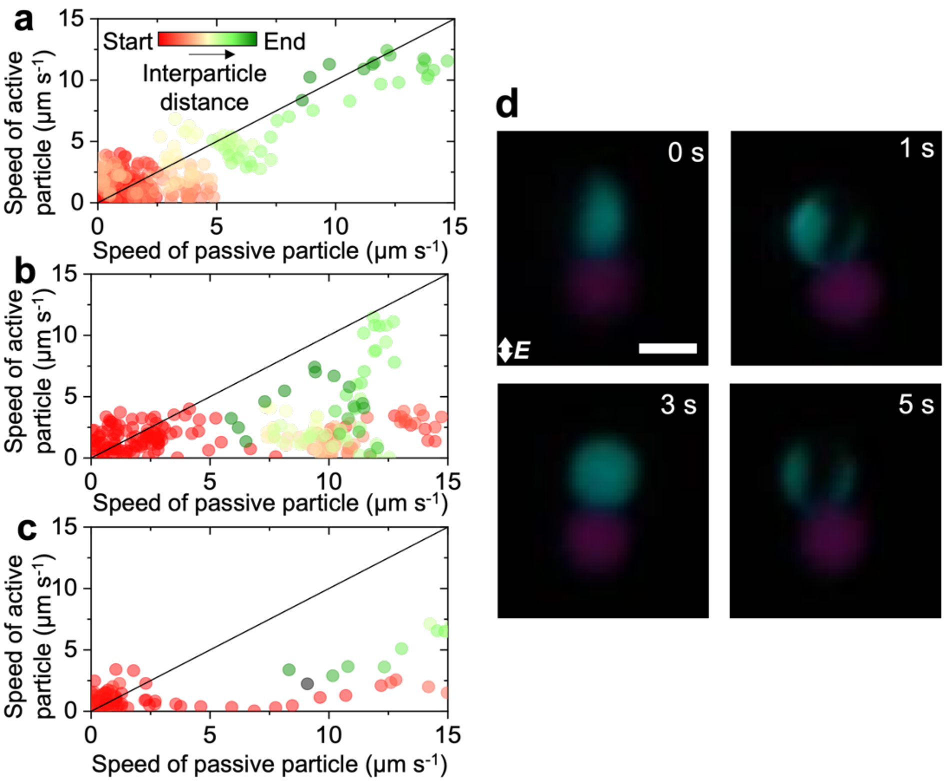

Figure S3. Covariance of the absolute values of speeds for each member of a pair of interacting particles, color-coded with respect to the interparticle distance. A line of slope 1 is superimposed on each covariance plot. (a) The speeds of two interacting passive particles show zero covariance while they are distant and show high covariance as they approach each other mutually. (b) The speeds of the linearly-propelled and passive particles show zero covariance while they are distant and weak covariance as they approach each other. (c) The speeds of the active and passive particles display zero covariance while they are distance and weak covariance as they approach each other. (d) Fluorescent microscope images of a pair doublet cluster showing the reconfiguration of the active particles on the surface of the passive particle. Scale bar in (d): 5 μm.

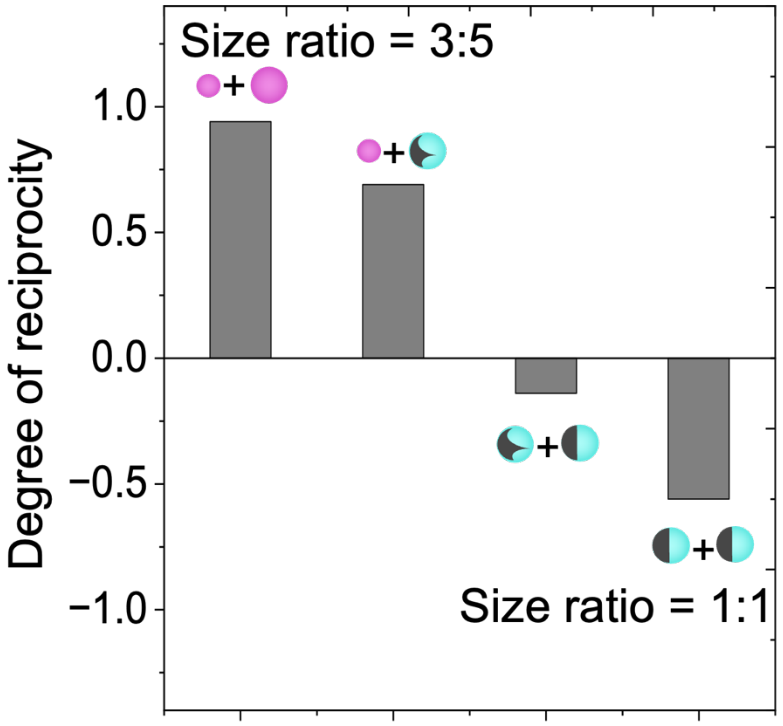

Figure S4. Degree of reciprocity of different passive-active particle pairs with varied particle and/(or) patch size. Passive particles are colored magenta and active particles are colored cyan. Interaction between two passive particles of different sizes is reciprocal, a passive and active particle of different sizes is non-reciprocal, and active particles of the same size and/or different patch sizes is non-reciprocal.

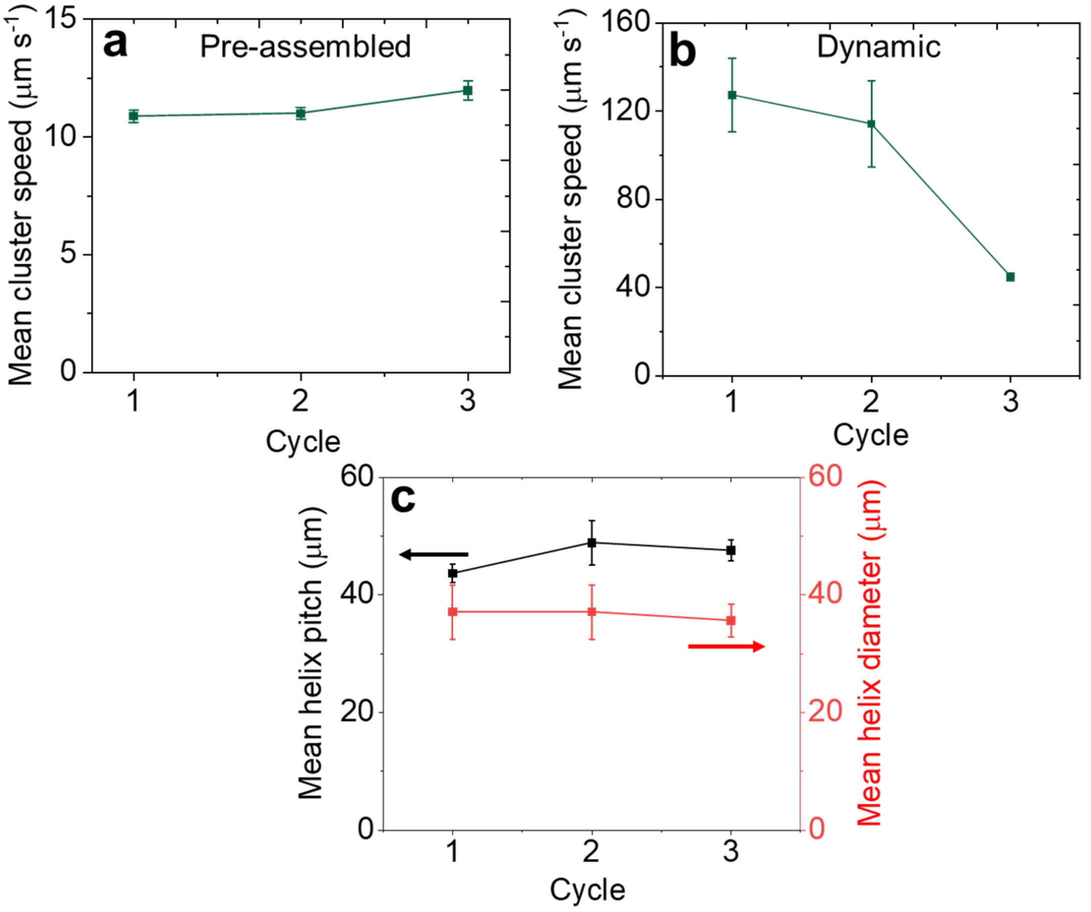

Figure S5. Cyclic retention and changes in pre-assembled and dynamic doublets respectively. (a) Similar mean cluster speed of a pre-assembled doublet in every field ON-OFF cycle. (b) Major differences can be seen in the mean cluster speed of a dynamic doublet in each field ON-OFF cycle. (c) No significant differences were observed in the helix pitch and diameter in a pre-assembled doublet for every field ON-OFF cycle. Error bars represent the standard deviation of at least 3 measurements taken for each cycle.

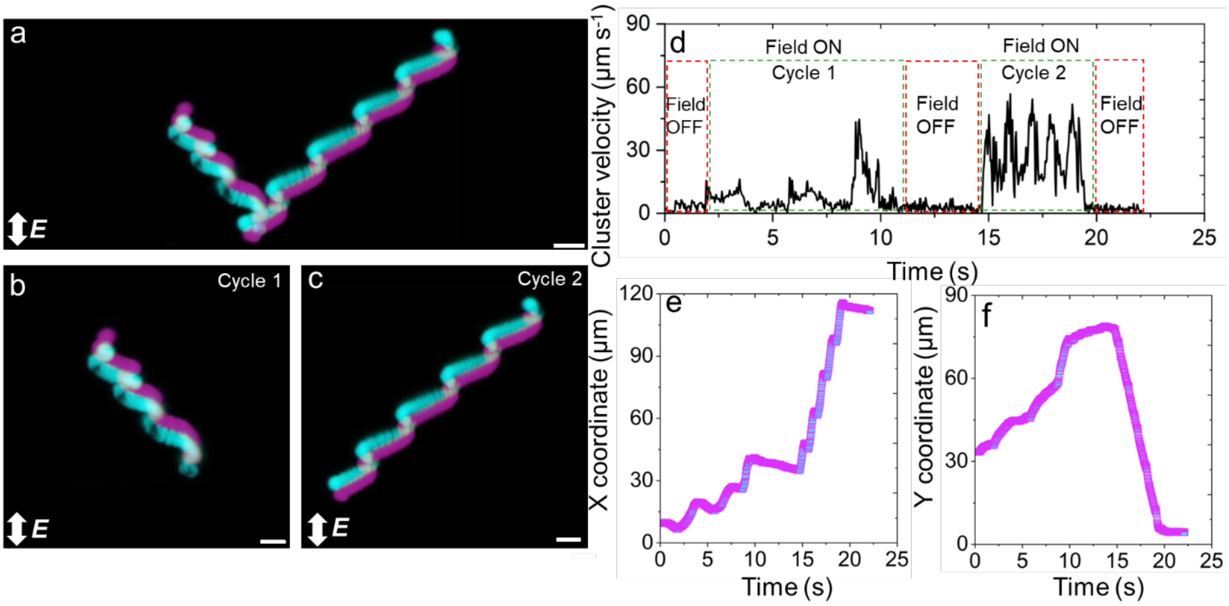

Figure S6. Motion of a dynamic metastable doublet for 2 field ON-OFF cycles. Scale bar in a, b and c is 10 μm. (a) Overlay of fluorescence microscopy images recording the motion of a dynamic metastable doublet as the external ac electric field is toggled on and off for 2 cycles. (b) Overlay of fluorescence microscopy images recording the motion of a dynamic metastable doublet for cycle 1 and (c) cycle 2. The cluster is assembled by the field, disassembles in its absence and reassembles on turning the field. (d) The active velocity of the cluster differs as the field is toggled on and off, and its trajectory varies in (e) the x and (f) y dimensions upon each disassembly/assembly cycle.

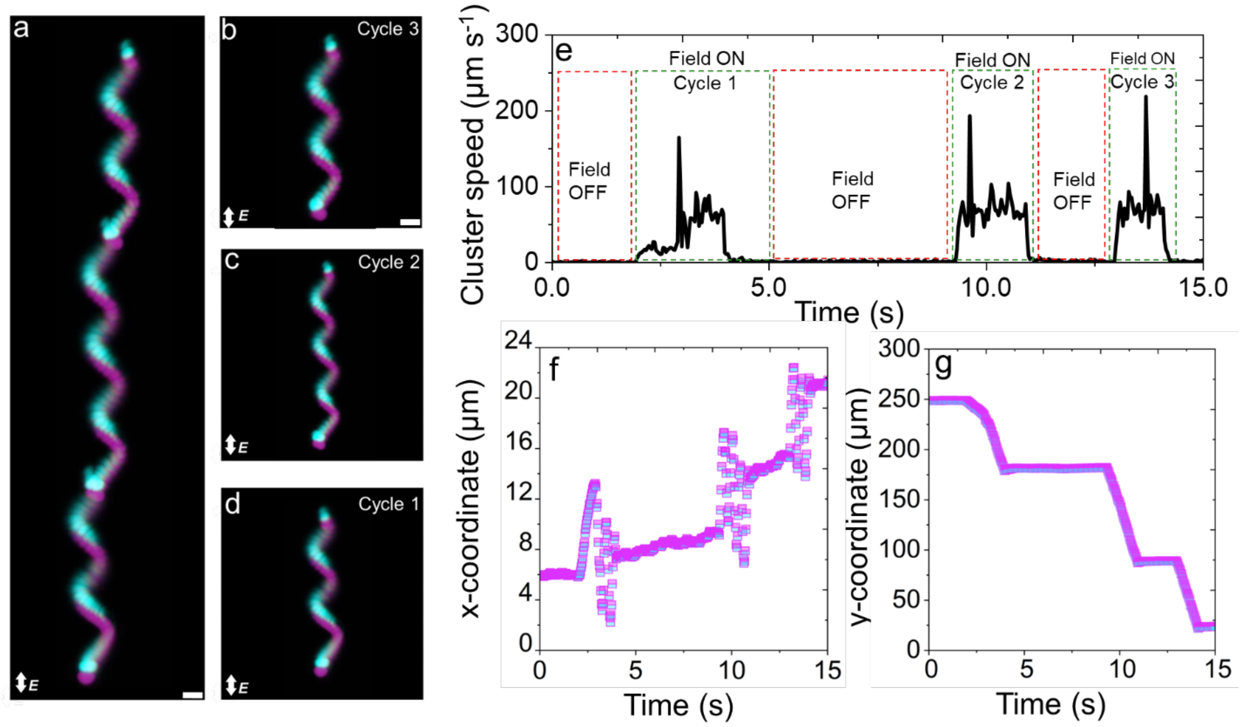

Figure S7. Motion of a pre-assembled stable doublet for 3 field ON-OFF cycles. Scale bar in a and b is 10 µm. (a) Overlay of fluorescence microscopy images recording the motion the doublet as the external ac electric field is toggled on and off for 3 cycles. (b) Overlay of fluorescence microscopy images recording the motion of a dynamic metastable doublet for cycle 1, (c) cycle 2, and (d) cycle 3. The cluster is pre-assembled without the field, and the assembly persists even after turning the field off. (e) The active velocity of the cluster remains nearly similar as the field is toggled on and off, and its trajectory remains nearly identical in (f) the x and (g) y dimensions throughout the 3 on/off cycles of the external field.

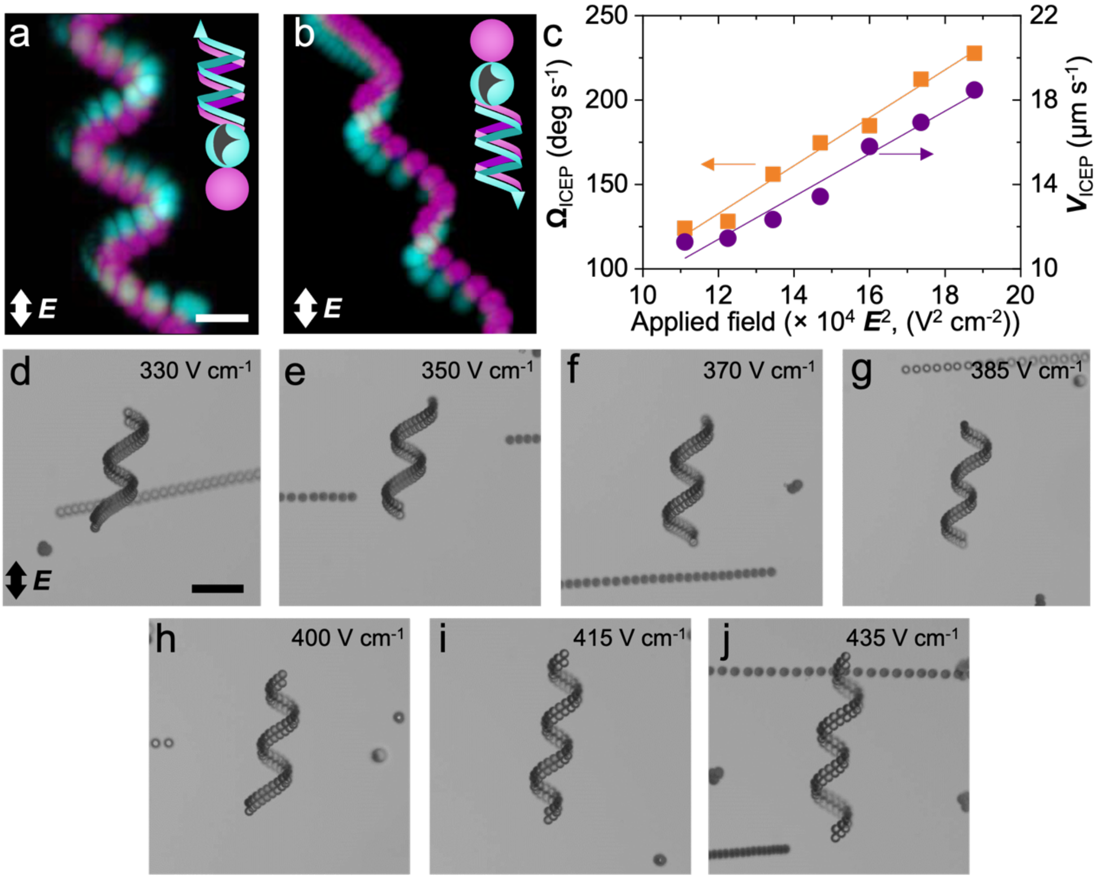

Figure S8. Motion and reconfiguration of small motile clusters. (a, b) Overlay of fluorescence microscopy images showing the motion of an assembled pair composed of an active (cyan) and a passive (magenta) particle. The insets are schematics highlighting the helical motion of the isolated pair along the axis where the active particle is present within the cluster, i.e., upward for (a) and downward for (b). (c) Linear dependence of rotational speed, $\boldsymbol{\Omega}_{\mathrm{ICEP}}$ (orange squares), and linear speed, V_{ICEP} (purple circles), on $\boldsymbol{E}^2$. The lines are linear fits to the experimental data. Scale bar in (a): 10 µm. (d-j) Overlays of bright field micrographs showing the motion of an assembled active-passive doublet cluster moving in helical trajectory via ICEP at increasing ac electric field strength $\boldsymbol{E}$. Each overlay shows motion for 5 seconds highlighting the increase in translational and rotational speeds with $\boldsymbol{E}$.

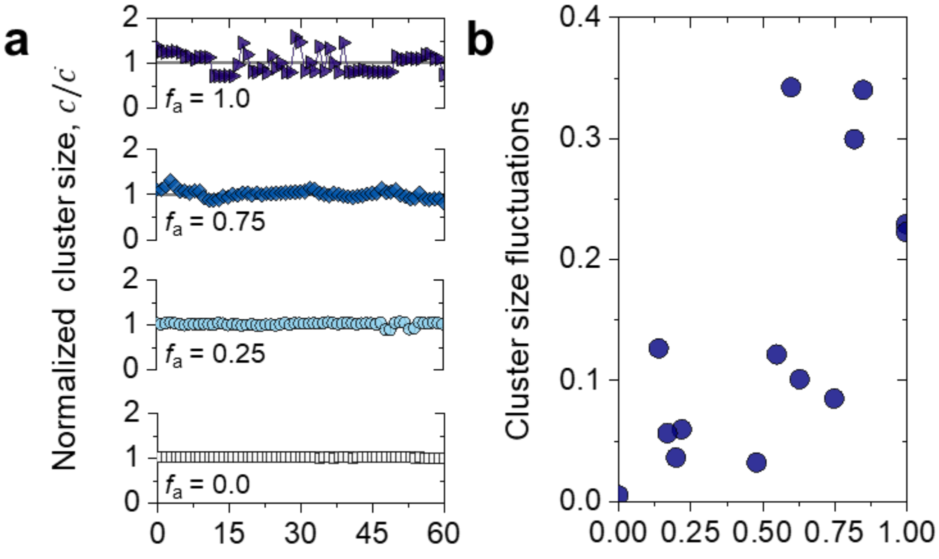

Figure S9 (a) Cluster size c normalized by the time-averaged cluster size $\bar{c}$. (b) Fluctuations in cluster size measured as the standard deviation of the data shown in (a). Clusters have larger size variations with increasing f_a.

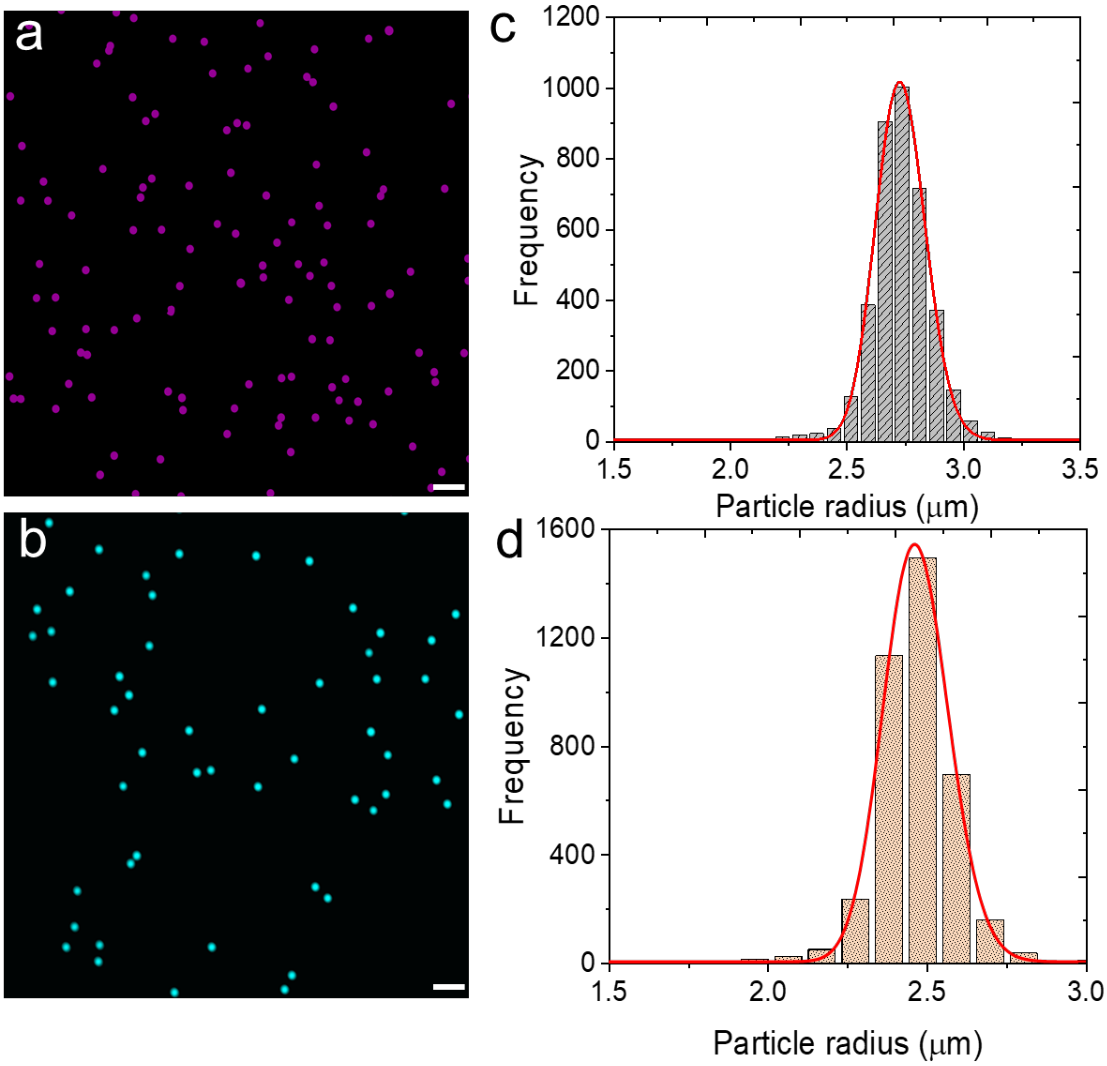

Figure S10. Particle size distributions. (a-b) Fluorescence micrographs of the polystyrene microspheres (Magsphere Inc.) used in the study and (c-d) the associated size distribution. The particles were labelled with (a) Fluorescein isothiocyanate and (b) Nile red, but shown in magenta and cyan, respectively, for visual accessibility. Scale bar: 20 μm. The bars in c and d are the measured frequencies of a given particle radius, and the lines represent the best fits using log-normal distribution function. The mean and standard deviation of the radius of the magenta and cyan particles respectively are 2.7±0.06 μm, and 2.6±0.04 μm.

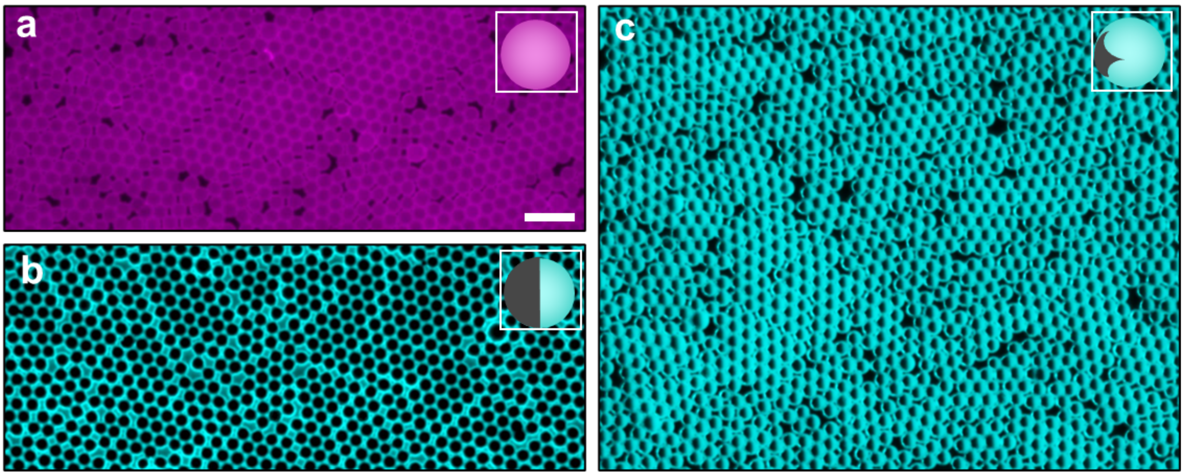

Figure S11. Fluorescence micrographs of monolayers of (a) isotropic passive particles, (b) Janus particles, and (c) triangular patched particles. The dark region on a particle represents the Au patch. Scale bar: 20 μm.

Caption for Video S1

Dynamics of individual passive and active particles in ac electric field (300 V/cm, 10kHz).

Caption for Video S2

Pair interaction dynamics using high speed microscopy (300 V/cm, 10kHz).

Caption for Video S3

Motion of dynamic metastable and pre-assembled stable doublets for various on/off cycles in ac electric field.

Caption for Video S4

Formation and motion of a helically propelling doublet in ac electric field (200 V/cm, 10kHz).

Caption for Video S5

Active motion of metastable clusters (300V/cm, 10kHz).

Caption for Video S6

Reconfiguration of large interacting metastable clusters via splitting and merging.

References

[1] H. Schmidle, S. Jäger, C. K. Hall, O. D. Velev, S. H. L. Klapp, *Soft Matter* **2013**, *9*, 2518.
[2] M. Z. Bazant, T. M. Squires, *Physical Review Letters* **2004**, *92*, 066101.
[3] S. Gangwal, O. J. Cayre, M. Z. Bazant, O. D. Velev, *Physical Review Letters* **2008**, *100*, 058302.
[4] J. G. Lee, A. M. Brooks, W. A. Shelton, K. J. M. Bishop, B. Bharti, *Nature Communications* **2019**, *10*, 2575.
[5] J.-Y. Tinevez, N. Perry, J. Schindelin, G. M. Hoopes, G. D. Reynolds, E. Laplantine, S. Y. Bednarek, S. L. Shorte, K. W. Eliceiri, *Methods* **2017**, *115*, 80.
[6] C. A. Schneider, W. S. Rasband, K. W. Eliceiri, *Nature Methods* **2012**, *9*, 671.
[7] J. G. Proakis, D. G. Manolakis, *Digital Signal Processing: Principles, Algorithms, and Applications.*, Prentice-Hall, Englewood Cliffs, NJ, **1996**.
[8] S. J. Orfanidis, *Introduction To Signal Processing.*, Prentice-Hall, Englewood Cliffs, NJ, **1996**.
[9] S. Kim, S. J. Karrila, in *Microhydrodynamics* (Eds.: S. Kim, S. J. B. T.-M. Karrila), Elsevier, **1991**, pp. 147–171.
[10] W. B. Russel, D. A. Saville, W. R. Schowalter, *Colloidal Dispersions*, Cambridge University Press, Cambridge, **1989**.
